# Efficiency Assessment between Entrapment and Covalent Bond Immobilization of Mutant β-Xylosidase onto Chitosan Support

**DOI:** 10.3390/polym15153170

**Published:** 2023-07-26

**Authors:** Gabriela Romero, Lellys M. Contreras, Carolina Aguirre Céspedes, Jeff Wilkesman, Josefa María Clemente-Jiménez, Felipe Rodríguez-Vico, Francisco Javier Las Heras-Vázquez

**Affiliations:** 1 Center for Environmental, Biological and Chemical Research, Experimental Faculty of Sciences and Technology, University of Carabobo, Valencia 2001, Venezuela; gvromero@uc.edu.ve (G.R.); wilkesman@lba.hs-mannheim.de (J.W.); 2 Department of Basic Sciences, School of Bioanalysis, Faculty of Health Sciences, University of Carabobo, Naguanagua 2005, Venezuela; 3 Department of Chemistry and Physics, University of Almeria, Building CITE 1, Carretera de Sacramento s/n, La Cañada de San Urbano, 04120 Almeria, Spain; jmclemen@ual.es (J.M.C.-J.); fvico@ual.es (F.R.-V.); 4 Centro de Energía, Department of Environmental Chemistry, Faculty of Sciences, Universidad Católica de la Santísima Concepción, Casilla 297, Concepción 4090541, Chile; caguirre@ucsc.cl; 5 Institute for Biochemistry, University of Applied Sciences Mannheim, Paul-Wittsack-Straße 10, D-68163 Mannheim, Germany; 6 Campus de Excelencia Internacional Agroalimentario ceiA3, University of Almeria, 04120 Almeria, Spain

**Keywords:** β-xylosidase, chitosan, enzyme immobilization, *G. stearothermophilus*, xylanase

## Abstract

The Y509E mutant of β-xylosidase from *Geobacillus stearothermophilus* (XynB2^Y509E^) (which also bears xylanase activity) has been immobilized in chitosan spheres through either entrapment or covalent bond formation methods. The maximum immobilization yield by entrapment was achieved by chitosan beads developed using a 2% chitosan solution after 1 h of maturation time in CFG buffer with ethanol. On the other hand, the highest value in covalent bond immobilization was observed when employing chitosan beads that were prepared from a 2% chitosan solution after 4 h of activation in 1% glutaraldehyde solution at pH 8. The activity expressed after immobilization by covalent bonding was 23% higher compared to the activity expressed following entrapment immobilization, with values of 122.3 and 99.4 IU.g^−1^, respectively. Kinetic data revealed that catalytic turnover values were decreased as compared to a free counterpart. Both biocatalysts showed increased thermal and pH stability, along with an improved storage capacity, as they retained 88% and 40% of their activity after being stored at 4 °C for two months. Moreover, XynB2^Y509E^ immobilized by covalent binding also exhibited outstanding reusability, retaining 92% of activity after 10 cycles of reuse. In conclusion, our results suggest that the covalent bond method appears to be the best choice for XynB2^Y509E^ immobilization.

## 1. Introduction

The use of enzymes to catalyze biotransformations represents a desirable alternative within the context of more eco-friendly conditions. However, in their native form, they are not always the best option compared to chemical catalysts. The use of enzymes can increase the cost of industrial processes; thus, reusable forms are gaining relevance and are being employed more frequently [1,2]. In this way, immobilization is a desirable alternative since it is usually accompanied by greater operational stability of the enzyme. In many cases, the immobilized enzyme significantly improves the pH and temperature range in which it can be used. This, coupled with the possibility of reusing the catalyst, increases the economic viability for the use of biocatalysts and makes it a desirable option in the transition of many industrial processes toward green chemistry [3]. 

Protein immobilization plays an important role in several areas. In life sciences and medicine, it forms the basis of many applications, such as biosensors, biomedical implants, recyclable biocatalysts, and protein arrays for drug screening, involving protein–protein or protein–ligand interaction [4]. In the chemical and food industry, it also plays a fundamental role, where the main reason for immobilization and, more recently, co-immobilization is reuse or continuous use within process reactors [5,6].

Methods for enzyme immobilization can be chemical and physicochemical [7], and their choice will depend on the nature of the support, the enzyme, and the application and use of the enzyme [8]. Enzymes can be immobilized on natural or synthetic supports through chemical means (binding them to supports by covalent bonds) or physical means (electrostatic, ionic forces, and membranes). They can be adsorbed and also trapped/encapsulated in some material through the addition of agents that form a protective film around the immobilized enzyme, allowing the selective passage of reagents and small-sized products [8].

The adsorption method, which is mainly based on physical adsorption or ionic binding, is a simple and reversible approach [9]. However, it can be challenging to find the conditions under which the enzyme maintains the bond strength, as well as its activity. On the other hand, entrapping enzymes within microporous gels, which are formed in the presence of the enzyme, prevents the enzyme from escaping the microporous structure while still allowing the entry and exit of substrates and products. This approach has been successfully tested in the pharmaceutical industry and other chemical processes and is known to enhance the mechanical stability of the biocatalyst [6,10]. In the case of covalent immobilization, the reaction occurs between amino acids on the enzyme surface and reactive groups placed on the support surface [11]. Immobilization of enzymes by covalent bonds using mesoporous silica and chitosan supports has been reported, resulting in improvements in the lifetime and thermal stability of the enzyme [10,12].

Chitosan, a poly-*N*-acetylglucosamine, is a modified oligosaccharide obtained by deacetylation of chitin, the second most abundant biopolymer after cellulose. Chitosan is insoluble in water, but in most organic acids, the amino group in the molecular chain is converted to ammonium ions, allowing solubility in the form of a gel that precipitates when the pH is increased, forming water-insoluble complexes. In this way, the gel forms spheres, membranes, capsules, and fibers, among others, which can be modified with treatments that improve stability and durability. Moreover, chitosan has the advantages of being biocompatible, biodegradable, nontoxic, nonantigenic, easy to obtain, and low cost, among others, and, at the same time, has unique characteristics of molecular structure, chemical, and biological properties [13,14]. One of the most common uses of chitosan is in the preparation of supports for enzyme immobilization [12,13,15,16,17]. In the literature, there are several reports about practical applications of chitosan after modifications with compounds such thiols [18], alginate [19], carrageenan, gelatin [20], and glutaraldehyde [21].

Enzymes with potential applications in biomass utilization processes become relevant in the promotion of the use of clean and renewable energy sources [22,23]. Such is the case of β-xylosidase from *Geobacillus stearothermophillus* (XynB2), which has been successfully overexpressed by recombinant technology and purified [24,25] and previously subjected to thorough biochemical [26,27,28] and biophysical characterization [25]. It has also been demonstrated that XynB2 can be transformed into a glycosynthase through site-directed mutagenesis of the catalytic nucleophile [29]. Additionally, the introduction of a new exo-xylanase activity into XynB2 was achieved by replacing tyrosine 509 with glutamic acid [30]. Notably, this mutant enzyme variant retained its xylosidase activity. These characteristics of XynB2 make it a highly promising candidate for industrial applications [31]. In this sense, the Y509E mutant of XynB2 (XynB2^Y509E^) has been immobilized in cross-linked enzyme aggregates (CLEAs) with remarkable improvements in its operational stability [32].

This present work addresses the importance of the selection of optimal kinds of immobilization with respect to the chitosan spheres, which were prepared using the mutant XynB2^Y509E^. Two protocols were implemented to immobilize the purified β-xylosidase mutant: by entrapment into chitosan beads and by the reinforcement of chitosan beads with glutaraldehyde. The second protocol was expected to promote covalent links with primary amino groups of XynB2^Y509E^. In this context, both protocols were evaluated through the immobilization conditions and kinetic properties of the free and immobilized mutant enzyme, intending to choose the more efficient catalyst. In addition, the storage stability and reusability of the catalysts were also determined. 

## 2. Materials and Methods

### 2.1. Materials

Materials used to make the spheres and to measure the enzyme activity like low molecular mass chitosan, acetic acid, glutaraldehyde (GTA), *p*-nitrophenyl-β-d-xylopyranoside (pNPX), and birchwood xylan were from Sigma (St. Louis, MO, USA). Acrylamide, bromophenol blue, and Coomassie R-250 brilliant blue used were from Fisher Scientific (Hampton, NH, USA). Bis-acrylamide, sodium dodecyl sulfate (SDS), glycerol, isopropyl β-d-*1*-thiogalactopyranoside (IPTG), molecular mass marker V849, and tris-base were from Promega (Madison, WI, USA). All other chemicals used were analytical grade reagents. 

### 2.2. Mutagenesis, Overexpression, and Partial Purification of XynB2^Y509E^

Site-directed mutagenesis of the *xynB2* gene was developed using the QuikChange II kit (Stratagene, La Jolla, CA, USA); the plasmid with the mutation was named pJAVI100 [32]. The *E. coli* C43 strain harboring the plasmid pJAVI100 was cultivated in LB broth supplemented with 100 µg/mL ampicillin at 37 °C, 250 rpm. When the optical density (600 nm) reached 0.5, the XynB2^Y509E^ production was induced with 0.1 mM of IPTG. After 18 h of induction at 37 °C and 250 rpm, the cells were harvested by centrifugation and lysed by sonication. The lysate was clarified by centrifugation at 10,000 rpm (Beckman Coulter J2-21, rotor JA20, Brea, CA, USA) for 30 min. The supernatant was collected and heat treated at 45 °C for 30 min and centrifuged again. The supernatant was dialyzed against 0.1 M citrate–phosphate–glycine buffer (CFG), pH 6.5 (29.41 g C_6_H_5_O_7_Na_2_.2H_2_O, 13.80 g NaH_2_PO_4_, and 7.51 g NH_2_CH_2_COOH in 1 L distilled water) at 4 °C overnight [32].

### 2.3. Preparation of Chitosan Spheres and Immobilization 

The spheres were prepared by the neutralization method [33], which consists of dripping the chitosan gel solution into an alkaline solution. Since chitosan is insoluble at basic pH, the drop solidifies because of polymer precipitation. Immobilization in chitosan was tested in two different ways:

*By entrapment*: 0.2 g of low-molecular-mass chitosan were dissolved in 10 mL of acetic acid (0.1 M) under continuous stirring at 120 rpm and with heating (60 °C). Four mL of the latest solution were added to 2 mL of the partially purified protein; the resulting suspension was added by dripping with a 27 G syringe into 200 mL of 0.5 M citrate–phosphate–glycine (CFG) buffer, pH 8.5 and 20% ethanol, forming spheres of approximately 2 mm in diameter. For hardening, spheres were stirred gently for 1 h in this solution. Then, the beads were extracted on a sieve and washed with CFG buffer, pH 6.5, reserving the buffer and wash water to verify the activity of the protein that was not incorporated into the spheres.

*By formation of covalent bonds on chitosan activated with glutaraldehyde*: spheres were prepared with 2% chitosan in acetic acid (0.1 M) and dripped into a solution of CFG buffer, pH 8.5 (0.5 M) and 20% ethanol, allowing maturation for 1 h (as described above). Then, the spheres were washed with distilled water and immersed in 1% glutaraldehyde solution for activation for 4 h. After that, the spheres were washed sufficiently to remove excess glutaraldehyde. For covalent protein binding, 0.3 g of wet spheres were incubated with 0.5 mL of protein partially purified by heating and 0.5 mL of CFG buffer pH 7.5 (0.1 M) under agitation of 200 rpm at 25 °C for 4 h. Then, the derivatives were filtered and washed with distilled water. After washing, the derivatives were stored in CFG buffer, pH 7.5 (0.1 M), at 4 °C until further use.

The yield of the XynB2^Y509E^ immobilization procedure was estimated by the difference between the protein measured from the starting amount (0.35 mg/mL) and the protein measured in the buffer solution used as a means for immobilization, plus the protein measured in the water of the 3 consecutive washes, as according to Equation (1):

(1) $$Protein\;immobilization\;yield\;\left(\%\right)=\frac{P_{0}-P\;}{P_{0}}\times 100$$

In immobilization by entrapment, P_0_ is the initial protein amount; P is the sum of the protein released during precipitation process and the protein released in the washes.

In immobilization on glutaraldehyde-activated chitosan spheres, P_0_ is the initial protein amount, and P is the protein in the supernatant after immobilization.

To calculate the expression yield of XynB2^Y509E^ after immobilization on chitosan by both methods, the following Equation (2) was used:

(2) $$Expression\;yield\;\left(\%\right)=\frac{Total\;activity\;of\;expressed\;enzyme\;\left(IU\right)\;}{\;Theoretical\;total\;activity\;of\;immobilized\;enzyme\;\left(IU\right)}\times 100$$

The term total activity of expressed enzyme refers to the total enzyme activity multiplied by the protein immobilization yield.

In order to evaluate more precisely the immobilization process, the percentage of activity recovery was calculated using the following Equation (3):

(3) $$Enzyme\;activity\;yield\;\left(\%\right)=\frac{Total\;activity\;of\;expressed\;enzyme\;\left(IU\right)\;\;\;}{Free\;enzyme\;activity\;\left(IU\right)}\times 100$$

### 2.4. Enzymatic Assays

*β-xylosidase activity*: β-xylosidase activity of free and immobilized enzyme was determined by measurement of *p*-nitrophenol (pNP) released from the substrate *p*-nitrophenyl β-d-xylopyranoside (pNPX) (Sigma). The reaction of immobilized XynB2^Y509E^ was initiated by the addition of 18 mg of beads with immobilized enzyme to 50 μL 2.2 mM substrate and 150 mM citrate-phosphate-glycine buffer at 50 °C. After 5 min, the reaction was stopped and color developed through the addition of 600 μL of 1M Na_2_CO_3_. Color intensity was read at 410 nm by using the extinction coefficient ∆ε = 18 mM^−1^ cm^−1^ [32] in an SP-830-plus spectrophotometer (Metertech). One enzyme unit (IU) was defined as the amount of enzyme required to produce 1 μmol of pNP per minute, and specific activity was defined as units per mg weight of protein-containing spheres. All assays were performed in triplicate and used as control spheres prepared according to the described technique without added protein. Protein determination was assayed by the Bradford method [34].

*Xylanase activity:* this activity was determined by measuring the amount of reducing sugars released from birchwood xylan employing the 3,5-dinitrosalicylic acid (DNS) method [35]. The reaction mixture consisted of 60 mg of spheres with immobilized enzyme and 0.9 mL of birchwood xylan at different concentrations in 0.1 M CFG buffer at pH 6.5. The enzymatic reaction was incubated for 1 h at 50 °C. After that, the reaction was stopped by adding 1 mL of DNS reagent, and tubes were incubated at 100 °C for 15 min. Subsequently, 2 mL of distilled water was added to the reaction tubes and mixed thoroughly. The absorbance of the liberated xylose was measured at 540 nm and the concentration was calculated by using the extinction coefficient ∆ε = 0.082 mM^−1^ cm^−1^ [32]. One unit of enzymatic activity was defined as the amount of enzyme required to release 1 µmol of reducing sugar xylose equivalents per minute. All assays were performed in triplicate and used as control spheres prepared according to the described technique without added protein. Protein determination was assayed by the Bradford method [34].

### 2.5. Optimization of Immobilization Processes 

In order to establish the optimal conditions during the immobilization by the entrapment process, the following parameters were investigated: chitosan concentration (1.0–5.0% *w*/*v*), type of acid (HCl as an inorganic acid and acetic acid as an organic acid) and its concentration (1 M and 0.1 M) for chitosan solubility, precipitant agents at two different final concentrations of 0.1 and 1 M (sodium and potassium hydroxide with pH values of 13 or 14 depending on their concentration, and CFG buffer at pH 8.5 and 9.5), and maturation time (1–4 h).

To determine the ideal conditions for the immobilization covalent process, the following parameters were investigated: enzyme concentration (0.753 and 0.389 mg/mL), glutaraldehyde concentration (1–5% *v*/*v*), cross-linking time (1–24 h), and immobilization time (1–24 h). 

All experiments were conducted in triplicate. The β-xylosidase activity under optimal conditions was defined as 100%.

### 2.6. Biochemical Characterization of Immobilized XynB2^Y509E^

*Effect of pH on the activity and stability of free and immobilized XynB2^Y509E^*: the optimum pH of free and immobilized enzymes was determined by incubating with CFG buffer ranging from pH 4.3–11 and keeping the same ionic strength (100 mM). To determine the pH stability, free and immobilized enzymes were incubated in CFG buffers at pH 4.3–11 for 1 h at 4 °C. The residual activity was measured using the standard chromogenic assay described above. Experiments for optimum pH and pH stability were performed in triplicate. 

*Effect of temperature on the activity and stability of free and immobilized* XynB2^Y509E^: the optimum temperature for both free and immobilized enzymes were determined by assaying enzymatic activity in 0.1 M CFG buffer (pH 6.5) at temperatures ranging from 40 to 80 °C for 5 min on p-NPX 2.2 mM as substrate. To investigate the thermal stability, the free and immobilized β-xylosidases were incubated in 0.1 M CFG buffer (pH 6.5) at different temperatures (30–90 °C) for 60 min. After that, the residual enzymatic activity was measured by the method described above. Experiments for optimum temperature and thermostability were performed in triplicate.

*Kinetic parameters of free and immobilized* XynB2^Y509E^: the kinetic constants (*K*_m_ and *V*_max_) for the free and immobilized enzymes were determined by measuring the enzymatic activity in a 200 mM CFG buffer (pH 6.5) at 50 °C with different substrate concentrations (0.22–2.00 mM). *K*_m_ and *V*_max_ were calculated from the hyperbolic adjustment using the Origin 8.0 program (Originlab Corporation, Inc., Northhampton, MA, USA). All assays were carried out in triplicate.

*Operational stability*: the retention of the immobilized enzyme activity was assayed under standard conditions for β-xylosidase activity for 10 consecutive cycles. After developing each reaction, the chitosan spheres with the immobilized enzyme were removed and washed with CFG buffer (pH 6.5) to eliminate any excess of substrate on the spheres. Then, the biocatalysts were re-used with fresh buffer and substrate in a new consecutive cycle. The β-xylosidase activity in the first cycle was considered as 100%.

*Storage stability*: the enzyme immobilized on chitosan spheres by entrapment and formation of covalent bonds was stored in CFG buffer at 4 °C for 2 months. At different times, free enzymes and biocatalysts were assayed for β-xylosidase activity. The relative β-xylosidase activity (%) was measured as a percentage of initial β-xylosidase activity (100%) considered as control. 

## 3. Results and Discussion

Within the general framework of obtaining more efficient biocatalysts, β-xylosidase XynB2^Y509E^ was immobilized onto chitosan spheres using two different methods. The effectiveness of both immobilization methods was evaluated by comparing the activity and stability of β-xylosidase when either trapped in or covalently attached to chitosan. 

XynB2^Y509E^ possesses dual activities, including its natural xylosidase activity, as well as a newly discovered exo-xylanase activity [30,32]. The introduction of a new catalytic function into the active site of XynB2 makes it an attractive candidate for studying various immobilization approaches. For industrial use, the enzyme must have high reusability, ease of recovery, and high operational stability. These properties can be acquired through enzyme immobilization. In general, immobilization results in an increase in operational stability, which ultimately results in a reduction in process costs [16,36].

### 3.1. Immobilization of XynB2^Y509E^ in Chitosan Spheres by Entrapment 

In this study, chitosan spheres were used as a model system for XynB2^Y509E^ immobilization. The first immobilization method performed was entrapment. In the stated method, a gel was formed in the presence of XynB2^Y509E^, and its subsequent precipitation resulted in the formation of spheres that contained the enzyme trapped within the chitosan matrix [37]. Ideally, in this type of immobilization, the enzyme retains the native structure as no strong modifications occur within the gel. Nonetheless, the primary drawback of this method is associated with the constraints on mass transfer that occur during an enzymatic cycle. Therefore, to achieve a successful entrapment, it is crucial to create a suitable environment within a porous material that enables unimpeded diffusion of the substrate and product, while limiting the mobility of the enzyme. The pore size of the spheres could also play a role in minimizing the potential adsorption of enzyme molecules on both the external surface and matrix of the support [2].

**Figure 1:**
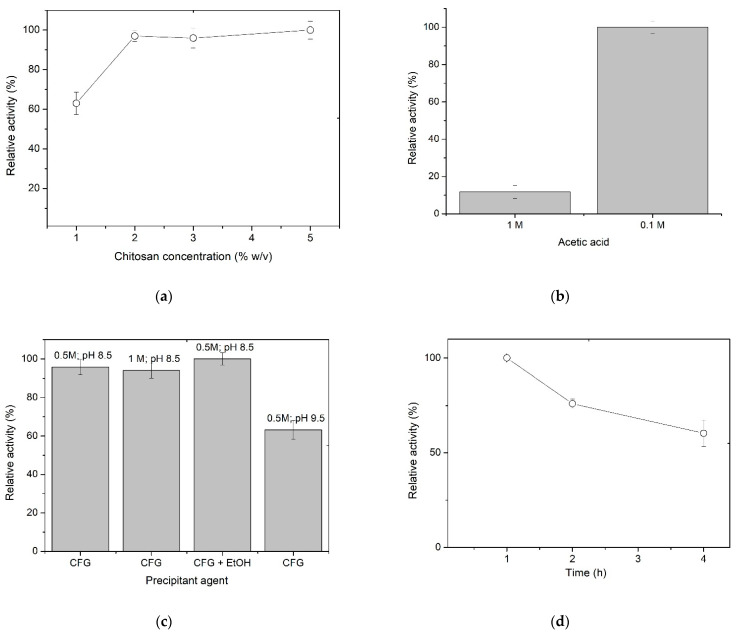
Standardization of the immobilization conditions of mutant XynB2^Y509E^ by entrapment in chitosan spheres. (**a**) Effect of chitosan concentration on β-xylosidase activity. (**b**) Effect of acid concentration for chitosan preparation. (**c**) Effect of concentration and pH of CFG buffer used as a precipitating agent on β-xylosidase activity. (**d**) Effect of the maturation time of the spheres prepared by alkali precipitation.

Several factors determine the optimal conditions for the preparation of chitosan spheres as a support for enzyme immobilization. In the case of immobilization by entrapment, one of the crucial factors is the concentration of chitosan used to prepare the spheres. Therefore, different concentrations of chitosan ranging from 1.0 to 5.0% (*w*/*v*) were studied in order to produce beads with better mechanical strength. Figure 1a shows that, at a very low concentration, the immobilization efficiency is lower because of enzyme leakage, which could be explained by a larger pore size in the spheres formed at a lower chitosan concentration. As the chitosan concentration increases, there is an improvement in immobilization efficiency, which may be due to the greater amount of groups available to form the porous cross-linking. The spheres elaborated with 5% chitosan also maintain a high relative activity, suggesting that the spheres prepared with chitosan concentrations between 2% and 5% have similar gel porosity and pore size. Consequently, these spheres exhibit similar diffusion of substrates and products. The lack of substantial difference in the activity of the enzyme immobilized at 2%, 3%, and 5% chitosan concentrations can be attributed to the negligible effect of natural polymers on the diffusion of small compounds into the spheres [38]. Although high chitosan concentrations favor entrapment, it was observed that there were mechanical difficulties in producing regular spheres from the 3% chitosan solution. Therefore, 2% was selected as the optimum chitosan concentration. 

Another important factor to consider during entrapment is the molecular mass of the chitosan used in the fabrication of the spheres. Sun and Zhang (2009) [39] have suggested that increasing the mass of chitosan, e.g., a polymer with longer molecular chains, would promote stronger spheres because of the increased interaction between them. Higher sphere strength is not always the ideal situation, as it has been observed that in high-molecular-mass chitosan spheres, the surface pore size is smaller. The smaller size prevents or restricts the passage of the substrate to the trapped enzyme, thus decreasing the efficiency of the biocatalyst. Based on the aforementioned, the chitosan utilized in this study had a low molecular mass (<190,000 Da).

During the optimization of the immobilization by entrapment, other factors were also evaluated, such as the type of acid and its concentration for a good chitosan solubility, the precipitating agent, and the maturation time of the spheres. Chitosan is soluble in solutions of different acids; thus, in this work, this polysaccharide was dissolved in two acids, a weak one, such as organic acetic acid [14,33], and a strong one, such as inorganic hydrochloric acid, at two different concentrations. As expected, the enzyme entrapped within the spheres formed upon dissolving chitosan in hydrochloric acid exhibited no activity (results not shown). At 1 M acetic acid, the activity of the entrapped enzyme was recorded to be 25% (Figure 1b). Significantly, when the concentration of acetic acid was decreased to 100 mM, the activity recovered from the spheres was 100%. The results can be explained in terms of the buffering capacity of chitosan. Acetic acid is known to have a pKa of 4.75 while the pKa of the primary amine of chitosan is around 6.5. Given the diluted nature of acetic acid (0.1 M), it can be inferred that both acetic acid and chitosan have a substantial buffering capacity. Moreover, the presence of a 25% acetyl content in the chitosan used for sphere preparation contributes to the buffering capacity of the acetic acid. Accordingly, our results suggest that the buffering capacity does not take place when the spheres are prepared in 1 M acetic acid. This inference finds support in the measurements of the free enzyme activity at different pH values [32]. 

The wild-type form of β-xylosidase XynB2 from *G. stearothermophilus* has been thoroughly characterized [24,25,26,27,28]. The β-xylosidase activity of the Y509E mutant of XynB2 has been previously characterized biochemically [30,32], presenting no major differences compared to the wild-type form, in relation to optimal conditions for enzyme activity and stability. From these works, it is known that, at the acid pH necessary to solubilize chitosan, the enzyme irreversibly decreases its activity over time. With this in mind, we can suggest that the absence of activity when chitosan is solubilized in hydrochloric acid is due to the inactivation of the enzyme by acid pH.

During beads preparation, solutions of sodium hydroxide, potassium hydroxide, and CFG buffer were evaluated to select the best precipitant agent. It was found that the enzyme trapped in the spheres precipitated in strong alkalis did not exhibit activity (results not shown). This could be because XynB2^Y509E^ loses its whole activity at a pH higher than 10, which is related to the ionic forms of the residues involved in the formation of the catalytic pocket [30,32]. Significantly, the enzyme entrapped in the spheres, which were precipitated using any of the four tested conditions of CFG buffer, remained active (Figure 1c). In this regard, 100% of recovered activity was obtained with the combination of CFG buffer and 20% ethanol. 

Finally, the last parameter analyzed was the maturation time of the spheres (Figure 1d). Results indicate that 1 h of maturation is the optimum time to recover 100% activity. Longer curing times, e.g., 2 or 4 h, decrease the activity by 25% or 40%, respectively. In summary, the entrapment immobilization of XynB2^Y509E^ on chitosan spheres was achieved by mixing the enzyme with 2% chitosan in 0.1 M acetic acid, followed by precipitating the beads using CFG buffer with ethanol and maturing them for 1 h. 

### 3.2. Imobilization of XynB2^Y509E^ on Chitosan Spheres by Covalent Bond Formation 

Since some enzymes can be inactivated by glutaraldehyde, a frequently used enzyme-support cross-linking agent, the concentration of this agent and the activation time were factors evaluated during the process of covalent immobilization of XynB2^Y509E^ on chitosan spheres. During the activation reaction of chitosan, glutaraldehyde facilitates the cross-linking of the polymeric chains, thereby improving the mechanical strength of the support and preventing its solubilization in an acidic medium because of its cationic nature [40]. Activation reaction also generates aldehyde groups on the chitosan surface that can react mainly with the amino groups of the enzyme, although they may eventually react with other functional groups such as thiols, imidazoles, or phenols [41]. Even though the mechanism of the reaction is not fully elucidated, it has been proposed that the formation of Schiff bases and nucleophilic substitutions may be involved [41,42].

**Figure 2:**
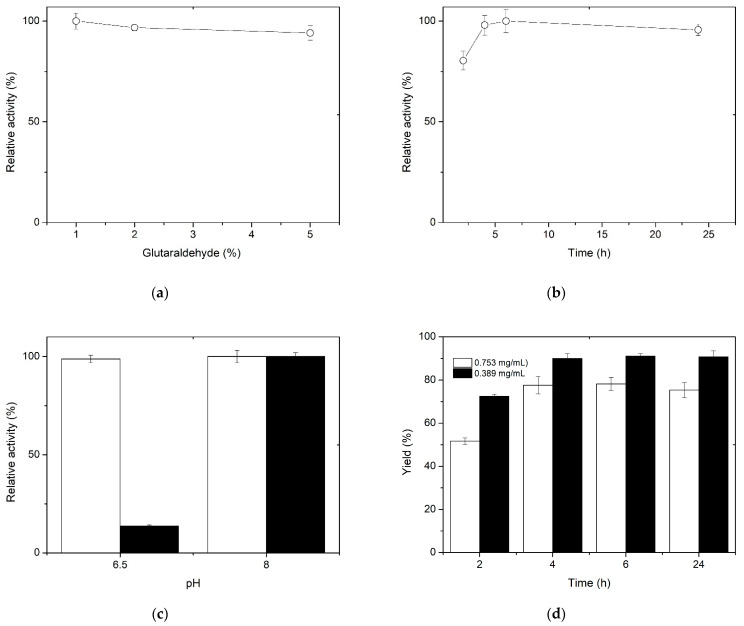
The effect of different preparation and activation conditions for chitosan spheres on β-xylosidase activity. (**a**) Effect of glutaraldehyde concentration on β-xylosidase activity. (**b**) Effect of reaction time for activation of chitosan spheres on β-xylosidase activity. (**c**) Effect of solution pH for chitosan sphere activation on β-xylosidase (white) and xylanase (black) activity of XynB2^Y509E^. (**d**) Yield according to protein concentration: 0.753 mg/mL (white), 0.389 mg/mL (black), and immobilization time.

Figure 2a shows that higher concentrations of glutaraldehyde resulted in decreased activity. It has been reported that in aqueous solution, glutaraldehyde can exist in its simplest form, a monomeric dialdehyde, but also as a dimer, trimer, and polymer [42]. As a result, the efficacy of glutaraldehyde to activate support could be rationalized with the multiplicity of structures. In this sense, Betancor et al. (2006) suggest that a polymerizing effect is also generated on the glutaraldehyde in solution, promoted by the increased reactivity of the molecule after the first amino–glutaraldehyde reaction [43]. Since 1% glutaraldehyde allows the recovery of 100% of the activity, this concentration was considered optimal. In relation to the activation time of the spheres with 1% glutaraldehyde, it was found that between 4 and 6 h preserve 100% of the activity. The results of Figure 2a,b allow us to conclude that the conditions of activation of the spheres for an optimum immobilization are 1.0% glutaraldehyde for 4 h.

Another factor evaluated during glutaraldehyde reaction with chitosan was the pH of the solution. The assay was run at two pH values (6.5 and 8), since it is known that at acidic pH values glutaraldehyde is in its monomeric form, which produces shorter bonds, while at more alkaline pH, larger polymers are formed, with the possibility of broader bonds [42]. Given that the XynB2^Y509E^ enzyme variant has the dual activities of β-xylosidase and xylanase, it was relevant to consider both pH conditions. Figure 2c shows that the activation of spheres at pH 6.5 and 8 preserves β-xylosidase activity, but there is a significant reduction in xylanase activity when sphere activation and enzyme binding occur at pH 6.5. This finding could be related to the difficulty of the substrate (xylan in the case of xylanase activity) to access the active site, as has been previously reported when XynB2^Y509E^ was immobilized as cross-linked enzyme aggregates [32].

After determining the optimal conditions for immobilizing XynB2^Y509E^ on activated chitosan spheres, the immobilization yield was evaluated according to Equation (1). Figure 2d shows the yields obtained for the four immobilization reaction times that were studied. The results reveal that the immobilization yield increases with longer reaction times, although the maximum yield obtained was 77%. In order to increase the immobilization yield, the amount of protein to be immobilized was reduced by half to 0.389 mg/mL, resulting in a 90% yield. The reason for the decreased yield when a higher enzyme concentration was employed can be attributed to the saturation of the support. The degree of deacetylation that influences the saturation degree ranges from 60 to 100% [44] in commercial applications and is ≥75% according to the manufacturer [45]. In this way, the enzyme concentration should be considered a crucial factor when using porous supports to immobilize enzymes [1,46].

Table 1 shows a comparison of the parameters that define the immobilization processes used to immobilize XynB2^Y509E^. The immobilization yield for XynB2^Y509E^ immobilized by entrapment was 68%, which is lower than the value obtained for covalent immobilization using the same enzyme load. This lower efficiency could be attributed to the chitosan used to prepare the spheres. Chitosan generally exhibits good performance in terms of entrapment efficiency. However, this efficiency is dependent on the molecular weight of chitosan. Alsarra et al. (2002) have reported that the use of high-molecular-weight chitosan leads to a higher enzyme loading [47]. Another parameter often used to determine the success of enzyme immobilization is the percentage of expression yield, which was calculated according to Equation (2). The values obtained, namely, 30% for immobilization by covalent bonds and 7.9% for immobilization by trapping, can be attributed to alterations in the microenvironment of the catalytic pocket, enzyme distortion, or limitations in diffusion caused by the immobilization process itself. The findings of this research indicate that the expressed activity of immobilized XynB2^Y509E^ is notably increased by activating the spheres using glutaraldehyde. 

Table 1 also shows the global enzyme activity yield, which is another parameter really pertinent to define the immobilization process [1]. According to Equation (3), the estimated enzyme activity yield for the entrapment immobilization was 38%, whereas for covalent immobilization using glutaraldehyde, it resulted in 67%. The loss of activity has also been found in other reports of enzyme immobilizations by the covalent bond formation, and it would be related to conformational changes in the structure as a result of covalent coupling [48]. 

Upon comparing the two methods, it was observed that the utilization of chitosan beads activated with glutaraldehyde significantly enhanced the efficiency of the immobilization process. Glutaraldehyde is a cross-linking agent with the ability to strengthen the enzyme structure and improve its binding to the support [41,42]. During the process of covalent immobilization, glutaraldehyde plays a dual role, acting as both a bead activator and a cross-linking agent for the chitosan. After support activation, chitosan spheres and enzyme molecules can also interact and react with each other. In fact, one aldehyde group of glutaraldehyde can react with the amino group of chitosan, while the other aldehyde group is available to form a covalent bond with XynB2^Y509E^ through its amino group. In this way, our results support the idea that the activated chitosan spheres behave as a multi-cross-linking agent, providing resistance and stability to the biocatalyst [12,49]. 

From Table 1, it is evident that the activity of the immobilized enzyme bound to chitosan was the highest. The lower activity of XynB2^Y509E^ after entrapment immobilization suggests that the enzymatic reaction catalyzed might be influenced by diffusional limitations. In conclusion, our results suggest that the covalent bond method appears to be the best choice for XynB2^Y509E^ immobilization.

### 3.3. Effect of Temperature and Thermostability

**Figure 3:**
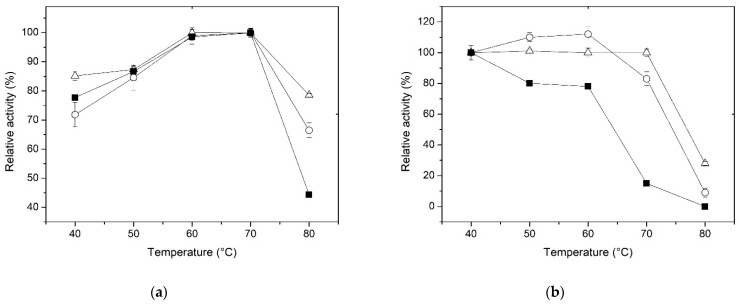
Effect of temperature on the β-xylosidase activity (**a**) and stability (**b**) of free XynB2^Y509E^ (■), XynB2^Y509E^ immobilized by entrapment on chitosan spheres (○), and XynB2^Y509E^ immobilized on glutaraldehyde-activated chitosan spheres (Δ).

The temperature effect on the β-xylosidase activity of the free and immobilized enzyme using the two proposed methods was determined in 0.1 M CFG buffer within the range of 40 to 80 °C. The results presented in Figure 3a show that the optimum temperature for both free and immobilized enzymes is around 70 °C. The fact that the optimum temperature for the activity of the immobilized enzyme, whether through entrapment or covalent binding, did not shift to higher values could suggest that the activation energies for the immobilized forms should be highly similar to that of the free enzyme. Although there is no change in the optimum temperature, it is worth noting that, at 80 °C, the enzyme—regardless of the immobilized method—exhibits a higher percentage of activity compared to the free enzyme (Figure 3a). These results suggest that the conformational flexibility of XynB2^Y509E^ was influenced by both immobilization methods. According to Munjal and Sawhney (2002), the use of a matrix to entrap enzymes can potentially cause hydrophobic and other secondary interactions, resulting in changes to the enzyme’s conformational flexibility. As a result, higher temperatures may be necessary to attain the correct conformation and maintain its reactivity [50]. Furthermore, Asgher et al. (2017a) proposed that the chitosan support can also protect the enclosed enzyme from the surrounding environment, effectively preventing denaturation that could potentially happen in the soluble form of the enzyme [51]. 

The increased rigidity of the enzyme resulting from covalent immobilization has also been reported previously. Roy et al. (1989) observed an improvement in the heat stability of β-glucosidase from *Myceliophthora thermophile* when it was immobilized on CNBr-activated sepharose [52]. Additionally, Abdel-Naby (1993) reported that the broader heat tolerance exhibited by covalently bound β-xylosidase and a xylanase to chitosan could be due to an increase in enzyme rigidity [53]. Furthermore, Figueira et al. (2011) reported that the immobilization of a fungal β-glucosidase in sol-gel results in an augmented heat tolerance [54]. More recently, a similar result was observed when the β-glucosidase from *Aspergillus niger* was immobilized trough covalent binding [48]. A possible explanation of the better heat-tolerance capacity of the XynB2^Y509E^ immobilized by the covalent method might be attributed to the formation of stronger bonds between the enzyme and the chitosan network. 

Thermostability was assessed by incubating the enzyme, whether free or immobilized, at temperatures ranging from 40 to 80 °C for 1 h in the absence of substrate, and then, the activity of the enzyme was determined at the optimal reaction temperature. The data regarding relative activities are displayed in Figure 3b. Between 50 and 60 °C, a slight increase in activity was observed in the enzyme immobilized by entrapment compared to the form immobilized on activated chitosan. This could be attributed to a phenomenon in which the pores of the chitosan sphere expand with rising temperature, facilitating better access of the substrate to the active site. At higher temperatures, however, the enzyme immobilized by covalent binding shows better stability. For example, at 70 °C, the free enzyme was quickly denatured, whereas the immobilized XynB2^Y509E^, whether by entrapment or covalent binding, retained approximately 80% and 100% of their activity, respectively. Furthermore, the relative activity of XynB2^Y509E^ immobilized on activated chitosan remains above 30% even after 1 h at 80 °C. Taken together, the results suggest that immobilization by entrapment or covalent binding leads to an increase in the thermal stability of XynB2^Y509E^, which makes these forms of the enzyme industrially more valuable than the free enzyme. To sum up, our findings are consistent with the notion that chitosan microspheres may offer protection to the enzyme against environmental factors that would otherwise impair its performance [51,55].

### 3.4. Effect of pH on Activity and Stability

**Figure 4:**
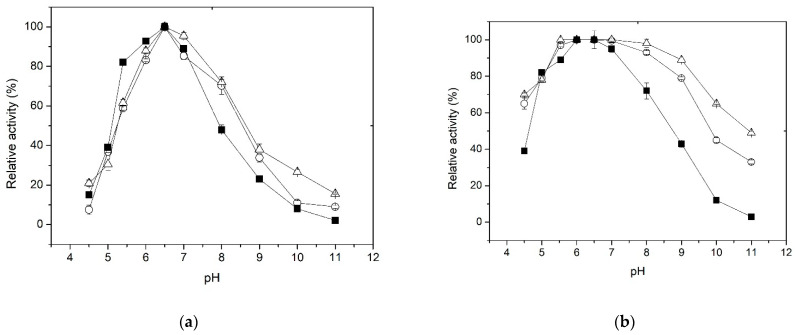
Effect of pH on the β-xylosidase activity (**a**) and stability (**b**) of free (■) and immobilized enzymes on chitosan by entrapment (○) and covalent bonds (△).

It is well known that variations in pH values can affect the formation and/or dissociation of the enzyme-substrate complex, hindering the formation of the catalytic pocket or active center of the enzyme and its stability. The effect of pH on enzyme activity, free and immobilized, is shown in Figure 4a. It is observed that the relative activity of the free and immobilized enzyme increases gradually in the pH range of 5.5 to 6.5, while the pH optima were almost the same (pH 6.5). At pH 7, the decrease in activity is more drastic in the free enzyme than in the two forms of immobilized enzyme. Furthermore, at pH 8, while the free enzyme retains 45% of activity, the immobilized forms retained 75% of their initial activity. Additionally, the pH-activity profiles indicated that XynB2^Y509E^, when entrapped within chitosan or covalent bounded to chitosan, were less susceptible to pH changes compared to the free enzyme. This effect can be attributed to the buffering properties of chitosan, which bears a reported p*K*_a_ value of 6.2–6.6 and acts as a buffer within the pH range of 5.5 to 7.4 [56,57]. 

Regarding the effect of pH on the stability of the free and immobilized enzyme, it was found that immobilization results in greater chemical stability of the enzyme at pH values from 7 to 11 (Figure 4b). For example, after incubation at pH 9 for 1 h, the free enzyme retains only 40% of its activity. In contrast, the enzyme forms immobilized by entrapment or covalent bonding retain 80 and 90% of their original activity, respectively. The improved alkaline tolerance capacity of XynB2^Y509E^ immobilized either by entrapment or covalent binding could be potentially attributed to the electrostatic interactions formed between the enzyme molecule and the chitosan matrix or network. These interactions could facilitate the maintenance of the biologically active conformation of the enzyme, thereby resulting in improved resistance to environmental changes [58]. The broad pH tolerance exhibited by XynB2^Y509E^ after chitosan immobilization makes it highly valuable for industrial applications. 

### 3.5. Effect of Immobilization on Kinetic Parameters

The kinetic parameters of the reaction of the free enzyme and both immobilized forms are compiled in Table 2. The K_m_ value for free XynB2^Y509E^ was found to be 0.9 mM, which aligns with findings from previous studies [30,32]. The XynB2^Y509E^ immobilized through entrapment on chitosan beads displayed an apparent K_m_ value of 0.7 mM, whereas the immobilized enzyme via the covalent method exhibited the same K_m_ value as the free enzyme (K_m_ = 0.9 mM). Since the K_m_ values were similar in magnitude, we can infer that the catalytic function of XynB2^Y509E^ was not significantly compromised by either of the two immobilization methods. On the other hand, enzyme immobilization caused a moderate decrease in V_max_, which might be caused by the diffusion limitation of substrate and product when XynB2^Y509E^ was entrapped within the chitosan sphere or to interaction of the enzyme with the functional groups on the surface of the chitosan beads in the covalent immobilization [58]. Other researchers have reported similar observations of a lower V_max_ following the immobilization of fungal xylanolytic enzymes either by covalent binding on chitosan spheres or by adsorption on aluminum hydroxide particles [59,60]. More recently, it has been reported that covalent bonding immobilization of acetylcholinesterase on chitosan spheres also resulted in a decrease in V_max_. This decrease was attributed to the restricted mobility of the enzyme, which could potentially induce alterations in its three-dimensional structure [61]. 

Catalytic turnover and catalytic efficiency (Table 2), calculated from *K*_m_ and *V*_max_ parameters, reflect the changes after immobilization. In general, the enzyme’s *k*_cat_ and catalytic efficiency (*k*_cat_/*K*_m_) decreased after immobilization. A significant decrease in turnover number (*k*_cat_) was observed for the two immobilized enzyme forms. Furthermore, the catalytic efficiency, *k*_cat_/*K*_m_, of the two immobilized enzyme forms was significantly lower than the value achieved by the free enzyme. The decrease in *k*_cat_/*K*_m_ was 420-fold for the enzyme immobilized by entrapment and 42-fold for the enzyme immobilized by covalent bonding. In other words, immobilization by covalent bonding showed a 10-fold improvement compared to immobilization by entrapment. Taken together, these results suggest a lower catalytic activity of the XynB2^Y509^ when it was immobilized. Several factors may contribute to the observed reduction in enzymatic activity: specifically, the diffusion of the substrate to the active site of the enzyme, the restricted mobility of the immobilized enzyme, and the partial denaturation of the enzyme during immobilization [58,59,60,61,62]. Although the catalytic efficiency of XynB2^Y509E^ declines with immobilization, the two immobilization methods have significantly improved the thermal and chemical stability of the enzyme. 

### 3.6. Operational and Storage Stability

**Figure 5:**
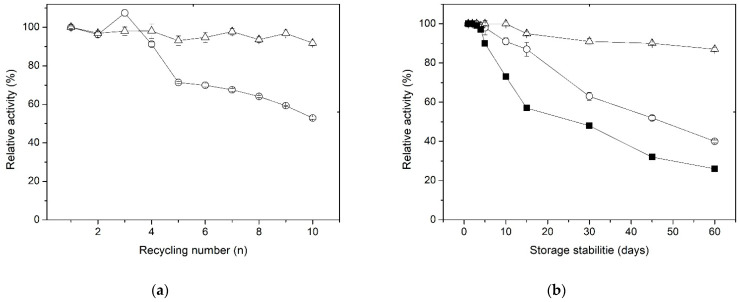
Operational stability of XynB2^Y509E^ immobilized on chitosan spheres. (**a**) Reusability of the immobilized enzyme by entrapment (○) and covalent bonds (△), measured through β-xylosidase activity under standard conditions. After each use, the spheres with the immobilized enzyme were removed from the reaction medium and sufficiently washed with CFG buffer to clean any amount of substrate and product that might be bound to the support. (**b**) Storage stability of the free (■) and immobilized enzyme on chitosan by entrapment (○) and covalent bonds (△) at 4 °C.

A key factor that reduces the overall cost of enzymes during industrial applications is the ability of immobilized enzymes to be regenerated and used continuously across multiple cycles [60]. The reusability of the XynB2^Y509E^ immobilized by entrapment and covalent bonding on chitosan spheres was assessed by conducting a series of 10 cycles (Figure 5a). It was observed that the XynB2^Y509E^ immobilized by entrapment preserves up to 65% of its activity after 5 cycles of reuse and more than 50% by the end of 10 reuse cycles. The observed decrease in activity for XynB2^Y509E^ entrapped in chitosan may be attributed to the enzyme leaking from the beads, as reported for other immobilized enzyme using entrapment methods [51]. This phenomenon was also supported by the determination of β-xylosidase activity in the washing water. The XynB2^Y509E^ immobilized through covalent linkages to chitosan retained 90% of its original activity after 10 cycles of reuse, indicating high and stable pNPX degradation performance in the repeated batches. This lower loss in activity can be attributed to the robust covalent attachment of XynB2^Y509E^ to chitosan spheres, which reduces the leaching of enzymes during catalysis. A similar result was observed by Pal and Khanum (2011) for xylanase immobilized on glutaraldehyde-activated alginate beads [63]. Although our results suggest that the immobilization of XynB2^Y509E^ on chitosan beads, whether through entrapment or covalent binding, exhibited favorable reusability, the biocatalyst immobilized on glutaraldehyde-activated chitosan beads emerged as a superior choice because of its higher reusability efficiency.

The long-term storage of industrial enzymes without any decline in biological activity has garnered significant attention in recent years. To study the impact of immobilization on the storage stability of XynB2^Y509E^, the free and immobilized forms of the enzyme mutant were stored at 4 °C for a duration of 60 days. The residual activity of the enzymes was assessed daily for the initial 5 days and subsequently at 15-day intervals (Figure 5b). The results show that the storage stability of the two forms of immobilized enzyme is substantially higher compared to the free enzyme. During the first 5 days, residual activity for all three forms was 100%. From the 5th day on, a noticeable decline in activity occurs in the free enzyme, reaching 50% after 15 days of storage at 4 °C. In contrast to the free enzyme, XynB2^Y509E^ immobilized by entrapment retained 85% of its initial activity, while the enzyme immobilized by covalent bonding retained 94% of its original activity. After a storage period of two months, it was observed that while the enzyme immobilized by entrapment gradually loses its catalytic stability, the enzyme covalently linked to glutaraldehyde-activated chitosan maintains its residual activity above 90%. This remarkable difference in storage stability suggests that the best immobilization for XynB2^Y509E^ is covalent binding. This finding is a desirable feature for the possible exploitation of this enzyme in industrial sectors that demand high storage stability.

## 4. Conclusions

When separated from their natural biocatalytic environment, enzymes tend to lose their activity or stability. This situation further underscores the importance of exploring new immobilization methods. In the present work, successful immobilization of a recombinant mutant of the family 52 glycoside hydrolase onto chitosan spheres was achieved using two different methods. The entrapment method resulted in an immobilization yield of 68%, whereas the covalent binding method achieved a yield of 90%. XynB2^Y509E^ immobilization on chitosan improved the stability properties to various parameters, such as pH, temperature, storage, and reuse, making it industrially useful. Altogether, our results suggest that the immobilization of xylanolytic enzymes on chitosan spheres is a valid and efficient approach. Significantly, this type of immobilization fulfills the requirement of being an environmentally friendly technique, and moreover, it enhances the long-term stability of both forms of the biocatalyst produced. Furthermore, the findings presented in this work contribute to the growing list of successful immobilizations using activated chitosan to immobilize enzymes. Finally, our findings also offer valuable insights into the optimization of supports and/or enzymes to enhance biocatalyst performance.

## Figures and Tables

**Table 1:** Immobilization yield and β-xylosidase activity of XynB2^Y509E^ trapped into chitosan spheres and covalently linked to glutaraldehyde-activated chitosan spheres.

Immobilization Parameter	Method	
	Entrapment	Covalent
PIY (%) ^a^	68	90
EY (%) ^b^	7.9	30
EAY (%) ^c^	38	67
AEI (IU.g^−1^ support) ^d^	99.4 ± 0.5	122.3 ± 1.3

^a^ PIY: immobilization yield in terms of immobilized protein. ^b^ EY: theoretical immobilized enzyme activity per mg of protein. ^c^ EAY: immobilization yield in terms of enzymatic activity. ^d^ AEI: activity measured in the biocatalyst after enzymatic immobilization.

**Table 2:** Kinetic parameters for the β-xylosidase activity of free and immobilized XynB2^Y509E^ on chitosan spheres.

XynB2^Y509E^	*K*_m_ (mM)	*V*_max_ (nmol/min)	*k*_cat_ (s^−1^)	*k*_cat_/*K*_m_ (s^−1^ M^−1^)
Free	0.9 ±0.1	1.1 ± 0.2	3.8	4.2
Covalent bond immobilization	0.9 ±0.1	1.0 ±0.3	0.1	0.1
Entrapment immobilization	0.7 ± 0.1	0.3 ± 0.1	0.01	0.01

## Data Availability

The data presented in this study are available on request from the corresponding author.
